# Discovering the Effects of Fisetin on NF-κB/NLRP-3/NRF-2 Molecular Pathways in a Mouse Model of Vascular Dementia Induced by Repeated Bilateral Carotid Occlusion

**DOI:** 10.3390/biomedicines10061448

**Published:** 2022-06-19

**Authors:** Marika Cordaro, Ramona D’Amico, Roberta Fusco, Alessio Filippo Peritore, Tiziana Genovese, Livia Interdonato, Gianluca Franco, Alessia Arangia, Enrico Gugliandolo, Rosalia Crupi, Rosalba Siracusa, Rosanna Di Paola, Salvatore Cuzzocrea, Daniela Impellizzeri

**Affiliations:** 1 Department of Biomedical, Dental and Morphological and Functional Imaging, University of Messina, Via Consolare Valeria, 98125 Messina, Italy; cordarom@unime.it; 2 Department of Chemical, Biological, Pharmaceutical and Environmental Sciences, University of Messina, Viale Ferdinando Stagno D’Alcontres, 98166 Messina, Italy; rdamico@unime.it (R.D.); rfusco@unime.it (R.F.); aperitore@unime.it (A.F.P.); tgenovese@unime.it (T.G.); linterdonato@unime.it (L.I.); frnglc96h11f112o@studenti.unime.it (G.F.); alessiaarangia@gmail.com (A.A.); salvator@unime.it (S.C.); dimpellizzeri@unime.it (D.I.); 3 Department of Veterinary Sciences, University of Messina, Viale Annunziata, 98168 Messina, Italy; egugliandolo@unime.it (E.G.); rcrupi@unime.it (R.C.); 4 Department of Pharmacological and Physiological Science, Saint Louis University School of Medicine, Saint Louis, MO 63104, USA

**Keywords:** dementia, fisetin, oxidative stress, inflammasome, NRF-2, NF-κB

## Abstract

Vascular dementia (VaD) is the second leading cause of dementia. The majority of VaD patients have cognitive abnormalities, which are caused by cerebral hypoperfusion-induced ischemia, endothelial dysfunction, oxidative stress, and neuroinflammation. Natural products are receiving increasing attention for the treatment of neuroinflammatory diseases. The aim of this study was to investigate the molecular pathways underlying the protective effects of fisetin, a flavonoid present in many fruits and vegetables, in a mouse model of VaD induced by repeated ischemia-reperfusion (IR) of the total bilateral carotid artery. Here, we found that VaD caused brain injury, lipid peroxidation, and neuronal death in the hippocampus, as well as astrocyte and microglial activation, and reduced BDNF neurotrophic factor expression together with behavioral alterations. In addition, VaD induced the activation of inflammasome components (NLRP-3, ASC, and caspase 1), and their downstream products (IL-1β and IL-18) release and promote activation of apoptotic cell death. Fisetin attenuated histological injury, malondialdehyde levels, inflammasome pathway activation, apoptosis, as well as increased BDNF expression, reduced astrocyte, microglial activation, and cognitive deficits. In conclusion, the protective effects of fisetin could be due to the inhibition of the ROS-induced activation of NF-κB/NLRP3 inflammasome together with the activation of antioxidant Nrf2/HO-1, suggesting a possible crosstalk between these molecular pathways.

## 1. Introduction

Vascular dementia (VaD) is the second most frequent type of dementia. It is caused by vascular alterations that induce dysfunction and damage to the cerebral vasculature, leading to cerebral blood flow (CBF) disruption, brain injury, and eventually cognitive impairment and memory loss [1]. Endothelial dysfunction, glial activation, demyelination, and blood–brain barrier collapse are among the neurovascular dysfunctions seen in VaD patients [2]. Diabetes, high blood pressure, smoking, and high cholesterol are all risk factors for VaD [3,4]. After the carotid arteries are restricted, a considerable reduction in regional CBF results in oxygen and glucose deprivation. These processes induce neuroinflammation and oxidative stress, which are the primary causes of VaD [1,5]. When the balance of reactive oxygen species (ROS) and antioxidants is upset, oxidative stress occurs, and it is increasingly linked to VaD. When CBF is disrupted, the mitochondrial electron transport chain is interrupted, electron leakage occurs, and an interaction with oxygen produces ROS [6]. Redox homeostasis instability and increased oxidative stress trigger a cascade of events that includes inflammasome activation [7,8]. Interleukin IL-1, a strong cytokine that orchestrates inflammatory pathways, is likely to play a role in VaD [9]. Inflammasome formation and activation are the key molecular mechanisms for IL-1 generation [10]. While there is no evidence that the inflammasome complex is directly involved in VaD, a cytokine profile of plasma from VaD patients revealed that IL-1 is the most prevalent [11,12]. Several studies have also reported a link between Nrf2 activation and the NLRP3 inflammasome [13,14]. The Nrf2 pathway is thought to be crucial in the development of inflammatory and neurodegenerative disorders. The Nrf-2 system controls the production of inflammasome downstream genes, and the two complexes may interact physically. Because proteins expressed upon Nrf-2 activation detoxify ROS, and ROS is predicted to trigger the NLRP3 inflammasome, ROS represents a link between both pathways [13,14]. Both routes have also been linked to the NF-κB factor. NF-κB is essential for the priming of the NLRP3 inflammasome, as well as the induction of Nrf2 [13]. Animal models of VaD are necessary for understanding the pathogenesis of dementia and determining the efficacy of new treatments. Polyphenols, particularly flavonoids found in plant diets, have been shown to be effective antioxidants in vitro and to have a beneficial effect in numerous VaD models [15,16]. Fisetin (3, 7, 3’, 4’-Tetrahydroxyflavone) is found in high proportions in fruits (e.g., strawberries, apples, grapes, mangoes, permissions, and peaches) and vegetables (e.g., onions, tomatoes, and cucumbers) [17]. The highest concentration of fisetin was found in strawberries followed by apple and persimmon [18]. Fisetin has been the topic of research in current years due to its anticancer, anti-inflammatory, memory-enhancing, and neuroprotective properties [19]. Fisetin has also been discovered to have substantial antioxidant capabilities in membrane settings, and it has been proposed as a possible treatment agent for a variety of free radical-mediated disorders [20]. Previous studies also showed the neuroprotective effects of Fisetin in experimental models of dementia induced by hyperhomocysteinemia [19] as well as of occlusion of the middle cerebral artery (MCAO) [21]. It was well known that the antioxidant effects of fisetin could be attributable to the activation of Nrf2/HO-1 [22]. However, Fisetin also blocked NLRP3 inflammasome activation and inhibited the release of IL-1β into the CNS in rats with encephalopathy [23]. Therefore, this study was planned to explore the protective effect of fisetin on VaD, pointing to the contribution of NF-κB/NLRP3 inflammasome and NRF2/HO-1 signaling pathways.

## 2. Materials and Methods

### 2.1. Animals

Sprague Dawley rats (males, 250 gr) and CD1 mice (males 25–30 gr) (Envigo, Milan, Italy) were housed in steel cages in a room at 22 ± 1 °C with a 12 h light/12 h dark cycle and were fed and watered regularly. The research was authorized by the University of Messina’s Animal Care Review Board. All animal tests complied with both Italian (D.Lgs 2014/26) and EU (EU Directive 2010/63) legislation.

### 2.2. Carrageenan (CAR)-Induced Paw Edema (Preliminary Data)

CAR-induced paw edema was performed as previously indicated by a subplantar injection of CAR (0.1 mL/rat of a 1% suspension in saline) (Sigma-Aldrich, Milan, Italy) into the right hind paw [24]. Increase in paw volume (ml) was measured using a plethysmometer (Ugo Basile, Varese, Italy) immediately prior to CAR injection and at 30 min and each hour for 6 h.

### 2.3. Experimental Groups (Preliminary Data)

First, we tested a possible dose response of fisetin effects in a classical acute model of inflammation as CAR-induced paw edema. Rats were divided into different groups:

- CAR + vehicle: rats were subjected to CAR-induced paw oedema, as described above, and administered orally with vehicle (1% aqueous solution of dimethyl sulfoxide (DMSO).
- CAR + Fisetin (5 mg/kg): same as the CAR + vehicle group, but Fisetin at dose of 5 mg/kg was administered instead of vehicle orally 30 min before CAR injection.
- CAR + Fisetin (20 mg/kg): same as the CAR + vehicle group, but Fisetin at dose of 20 mg/kg was administered instead of vehicle orally 30 min before CAR injection.
- CAR + Fisetin (40 mg/kg): same as the CAR + vehicle group, but Fisetin at dose of 40 mg/kg was administered instead of vehicle orally 30 min before CAR injection.
- Sham-operated groups received saline instead of CAR and were treated orally with vehicle or fisetin.

### 2.4. Animal Model of Repeated, Temporary Bilateral Carotid Occlusion

VaD induction was carried out in mice as previously stated by repeated, temporary bilateral carotid occlusion [25]. After anesthesia, the bilateral carotid arteries were ligated for 10 min and then unrestrained for 10 min, repeating the cycle three times. The incision was closed once the threading was removed. The mice were killed 15 days following induction, and their brains were taken and processed.

### 2.5. Experimental Groups

The animals were indiscriminately distributed into the following groups:

Group 1: Sham + vehicle = mice were exposed to the surgery without carotid arteries ligation and were treated orally with 1% aqueous solution of dimethyl sulfoxide (DMSO), (vehicle) once a day for 15 days (N = 12).

Group 2: Sham + Fisetin = same as the sham + vehicle group, but Fisetin at a dose of 40 mg/kg orally, dissolved in 1% aqueous solution of dimethyl sulfoxide (DMSO), was administered 24 h after the surgical procedure without carotid arteries ligation and once a day for 15 days thereafter (data not shown) (N = 12).

Group 3: VaD + vehicle = the mice were subjected to the VaD surgery described above and treated with vehicle as indicated for sham + vehicle group (N = 12).

Group 4: VaD + Fisetin = same as the VaD + veh group, but Fisetin at a dose of 40 mg/kg orally, dissolved in 1% aqueous solution of dimethyl sulfoxide (DMSO), was administered 24 h after the surgical procedure and once a day for 15 days thereafter (N = 12). The dose and the route of administration of Fisetin were chosen based on a previous study [26]. Because no significant difference was found between the sham + vehicle and sham + Fisetin, only data regarding the sham + vehicle groups were shown.

### 2.6. Behavioral Testing

Behavioral testing on all mice was conducted 1 day before and 15 days after VaD induction.

- Novel object recognition test (NOR): This model was performed as previously described [26]. The exploration time for the animal to investigate the new object was reported.
- Morris water maze (MWM): The MWM approach was used to investigate spatial learning and memory. In navigation trials (3 trials per day for 5 days), the mice’s escape latency (the time it took them to identify and stand on a platform under water) was assessed, and the frequency time around and within the platform quadrant was measured in probe trial (at 6th day) [26].

### 2.7. Light Microscopy

Histopathological examination with hematoxylin and eosin staining was performed as previously described [26,27,28,29]. The brains were collected rapidly and preserved in 4% formalin. A total of 7 μm slices were produced and colored after dehydration with ethanol and embedding with paraffin. The hippocampal CA1 and CA3 areas were then examined using a Leica DM6 microscope and Leica LAS X Navigator software (Leica Microsystems SpA, Milan, Italy). Ischemic neuronal damage was scored as previously [26]. All the histological studies were completed in blind.

### 2.8. Evaluation of Tissue Lipid Peroxidation

Malonaldehyde (MDA) levels were assessed at the end of the experiments. Briefly, after homogenization with opportune buffer, MDA absorbances were measured at 650 nm, using a spectrophotometer [30,31,32].

### 2.9. Immunohistochemical Localization of GFAP and Iba-1

Immunohistochemical analysis was performed as previously described [33,34]. The sections were incubated overnight with primary antibodies: with mouse monoclonal anti-GFAP and anti-Iba-1 (SCB; 1:200 in PBS, *v/v*). Sections were cleaned with PBS and then treated as indicated previously [35]. Five stained sections from each mouse were scored in blind and observed using a Leica DM6 microscope (Leica Microsystems SpA, Milan, Italy) following a typical procedure. The histogram profile was related to the positive pixel intensity value obtained [35].

### 2.10. Immunofluorescence for BDNF and NRF-2

Sections were incubated with the following primary antibodies: polyclonal anti-BDNF (SCB; 1:200 in PBS, *v/v*), or monoclonal anti- NRF-2 (1:50; SCB) as previously described [36,37]. Sections were washed with PBS and were incubated with secondary antibody TEXAS RED-conjugated anti-rabbit Alexa Fluor-594 antibody (1:1000 in PBS, *v*/*v* Molecular Probes, UK) and with FITC-conjugated anti-mouse Alexa Fluor-488 antibody (1:2000 *v*/*v* Molecular Probes, UK) for 1 h at 37 °C. Sections were rinsed and stained for nuclear signal with 4′,6′-diamidino-2-phenylindole (DAPI; Hoechst, Frankfurt; Germany) 2 μg/mL in PBS. Sections were observed and photographed using a Leica DM6 microscope (Leica Microsystems SpA, Milan, Italy).

### 2.11. Western Blots for Nuclear Factor NF-kB, NLRP-3, ASC, Caspase 1, NRF-2, HO-1, Bax, and Bcl-2

Cytosolic and nuclear extracts were prepared as previously described [35,38,39,40]. The following primary antibodies were used: anti-NF-κB (SCB; 1:500 #sc8008), anti-NRF-2 (sc-365949, 1:1000, SCB), anti-HO-1 (sc-136960, 1:1000 SCB), anti-Bcl-2 (SCB, sc-7382), anti-Bax (SCB, sc-7480), anti- NRLP3 (SCB, sc-66846), or anti-ASC antibody (SCB, N-15: sc-22514-R) or anti-Caspase-1 p20 (SCB, G-19: sc-1597) in 1 × PBS, 5% (*w*/*v*) non-fat dried milk, 0.1% Tween-20 at 4 °C overnight. Membranes were incubated with peroxidase-conjugated bovine anti-mouse IgG secondary antibody or peroxidase-conjugated goat anti-rabbit IgG (Jackson ImmunoResearch, West Grove, PA, USA; 1:2000) for 1 h at room temperature. Anti-β-actin or anti-lamin A/C antibodies were used as controls. The expression of protein bands was detected by a procedure previously described [35].

### 2.12. IL-1β and IL-18 Cytokines Levels

The IL-1β and IL-18 in homogenates of hippocampus tissues and serum were measured using IL-1β and IL-18 ELISA Kit, respectively. All ELISA assays were performed strictly according to the manufacturer’s instruction of kits (Thermo Fisher Scientific, Waltham, MA, USA) [41,42].

### 2.13. Terminal Deoxynucleotidyl Nick-End Labeling (TUNEL) Assay

Apoptosis was analyzed by a TUNEL assay using a cell death detection kit. TUNEL staining for apoptotic cell nuclei was performed as previously described [35,43].

### 2.14. Materials

All chemicals were analytical grade or higher. Fisetin (Fustel) was purchased from Selleckchem. Biogenerica CT, Italy.

### 2.15. Statistical Evaluation

The mean standard error of the mean (SEM) of N observations is used to calculate all findings. N is the number of animals. The photos for histology/immunohistochemistry were from at least three different experiments. The 0.05 *p* value was significant. A one-way ANOVA was used for multiple comparisons, followed by a Bonferroni post hoc test.

## 3. Results

### 3.1. Acute Effects of Fisetin on CAR-Induced Paw Edema: Preliminary Data

**Figure 1:**
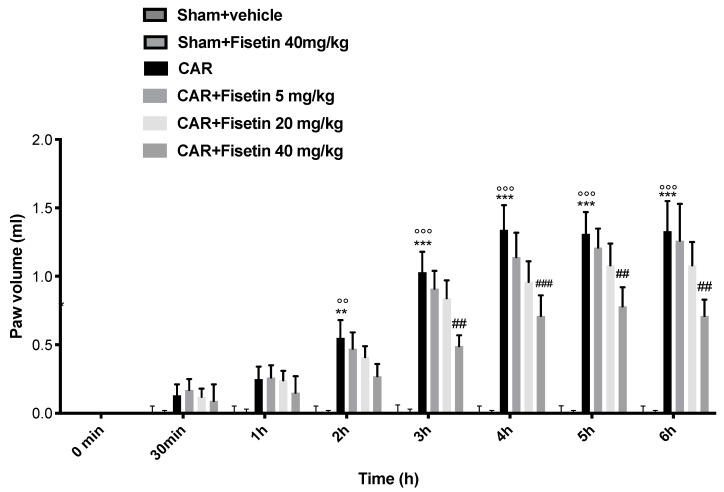
Preliminary data of anti-inflammatory effects of Fisetin on CAR-induced paw edema on different doses. The animals were treated at different doses, respectively, 5, 20, and 40 mg/kg. Values are means ± SEM of 6 animals for each group; *** *p* < 0.001 vs. sham + vehicle. ** *p* < 0.01 vs. sham + vehicle, °°° *p* < 0.001 vs. sham + Fisetin 40 mg/kg, °° *p* < 0.01 vs. sham + Fisetin 40 mg/kg. ### *p* < 0.001 vs. CAR. ## *p* < 0.01 vs. CAR.

To better choose the fisetin dosage and understand which dose of Fisetin could be efficacious, we preliminarily studied the acute effects of Fisetin on a classical model of inflammation, such as CAR-induced paw edema. We tested three different doses: 5, 20, and 40 mg/kg. The intraplantar CAR injection caused a critical time-dependent increase in the paw volume in the CAR-injected rats until 6 h compared to the shams. Fisetin at doses of 5 and 20 mg/kg was not able to significantly reduce the paw edema, while the higher dose of 40 mg/kg was able to reduce the paw inflammation starting from 3 h until 6 h post CAR. No paw edema increase was measured in both sham groups (Figure 1).

### 3.2. Fisetin Ameliorated Memory Deficits

**Figure 2:**
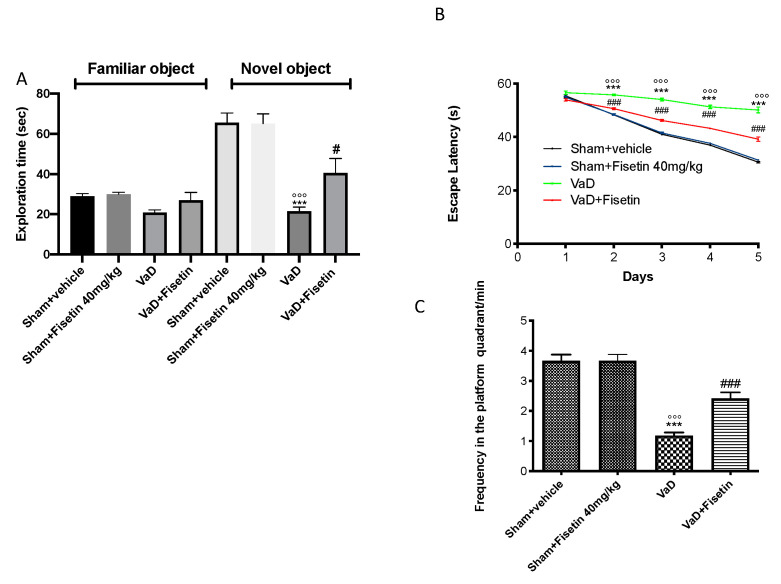
Fisetin administration on behavioral alterations after VaD induction. NOR and MWM tests were evaluated. (**A**) Exploration time in s; (**B**) escape latency; (**C**) frequency in the platform quadrant/min. Values = means ± SEM of 6 animals for each group; *** *p* < 0.001 vs. sham + vehicle, °°° *p* < 0.001 vs. sham + Fisetin 40 mg/kg. # *p* < 0.05 vs. VaD. ### *p* < 0.001 vs. VaD.

To assess the cognitive purpose, we performed the NOR test. During the exercise, there was no visible difference in the time expended discovering novel objects between the controls and VaD animals. The VaD mice showed significantly inferior curiosity in the novel object 15 days after injury (Figure 2A), designating a modification in cognitive function; nevertheless, the exploration time for the NOR was amplified in mice treated with Fisetin compared to the vehicle groups (Figure 2A). The MWM test was achieved to appraise the effect of Fisetin on memory harms. The period to discovery of the platform during training was increased in the VaD-subjected animals compared to the controls (Figure 2B). The Fisetin diminished the escape latency (Figure 2B). In addition, the frequency time across and inside the target quadrant of the platform during the probe trial was reduced in the VaD + vehicle animals (Figure 2C). The Fisetin treatment augmented the frequency time, enhancing the cognitive deficits (Figure 2C).

### 3.3. Fisetin Reduced Histological Parameters

**Figure 3:**
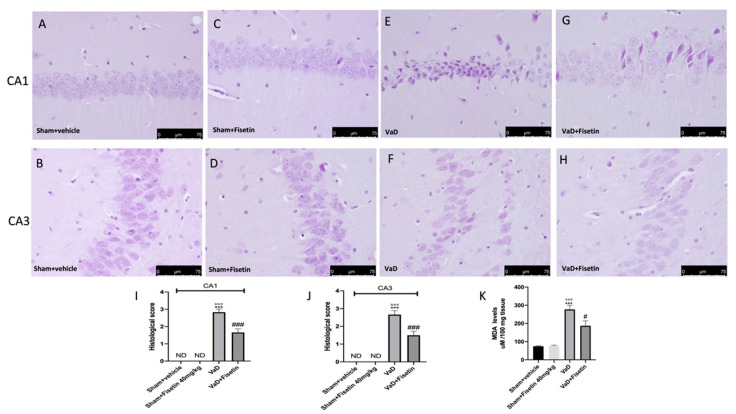
Fisetin administration on VaD induced histological damage and lipid peroxidation. Histological assessment in CA1 and CA3 regions, respectively, (**E**,**H**): (**A**,**B**) sham + vehicle group, (**C**,**D**), sham + Fisetin, (**E**,**F**) VaD + vehicle group, (**G**,**H**) VaD + Fisetin group, (**I**,**J**) histological scores. Figures are from at least three divided experiments. (**K**) MDA levels. Values are means ± SEM of 6 animals for each group. *** *p* < 0.001 vs. sham + vehicle, °°° *p* < 0.001 vs. sham + Fisetin 40 mg/kg. # *p* < 0.05 vs. VaD, ### *p* < 0.001 vs. VaD. Scale bar: 75 μm. Magnification (40×).

All slices were marked with H&E to detect the gravity of the damage in the hippocampus regions 15 days following injury. The hippocampus of the controls had a regular structure (Figure 3A–D; hippocampal CA1 and CA3 regions, and relative histological analysis Figure 3I,J. Disorderly and strictly stained neurons were observed in the hippocampal areas of the mice subjected to the VaD injury (Figure 3E,F, hippocampal CA1 and CA3 and relative histological analysis Figure 3I,J). The brain sections from the VaD mice treated with Fisetin displayed an obvious restructuring of the hippocampal CA1 and CA3 regions, with an enlarged number of hippocampal neurons (Figure 3G,H, hippocampal CA1 and CA3 regions and relative histological analysis Figure 3I,J).

### 3.4. Fisetin Reduced Lipid Peroxidation

To evaluate oxidative stress, the MDA levels, indicators of lipid peroxidation, were measured. Increased hippocampal MDA levels were observed in the mice subjected to VaD (Figure 3K). Fisetin reduced the MDA levels (Figure 3K).

### 3.5. Fisetin Reduced Neuroinflammatory Markers Expression and Increased BDNF Expression

**Figure 4:**
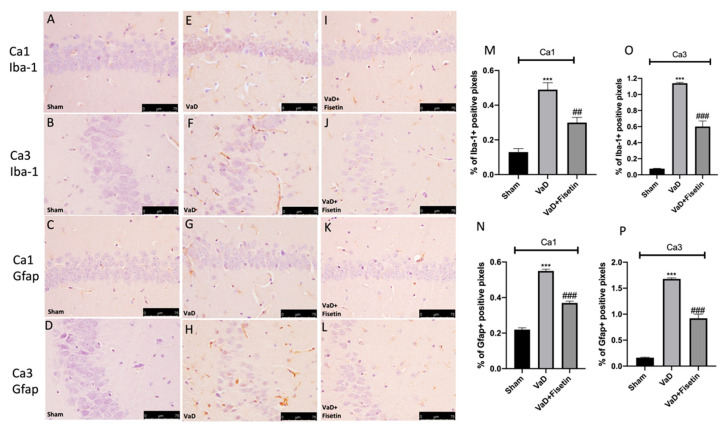
Fisetin administration on astrocyte and microglial activation. Immunohistochemistry for GFAP and IBA-1 in CA1 and CA3 regions, respectively, (**A**–**D**) sham group, (**E**–**H**) VaD + vehicle group, (**I**–**L**) VaD + Fisetin group. The results are expressed as % of positive pixels (**M**–**P**). Figures are representative of at least three independent experiments. Values are means ± SEM of 6 animals for each group. *** *p* < 0.001 vs. sham, ### *p* < 0.001 vs. VaD, ## *p* < 0.01 vs. VaD. Scale bar: 75 μm. Magnification (40×).

To estimate the effect of Fisetin on neuroinflammation, we assessed astrocytes (GFAP) and microglia (Iba-1) markers by immunohistochemistry. Amplified positive staining for the GFAP and Iba-1 were perceived in the VaD vehicle animals (Figure 4E–H and see graphics in Figure 4M–P) compared to the sham animals (Figure 4A–D and see graphics in Figure 4M–P). Fisetin was able to moderate immunoreactivity for the GFAP and Iba-1 (Figure 4I–L and see graphics in Figure 4M–P).

**Figure 5:**
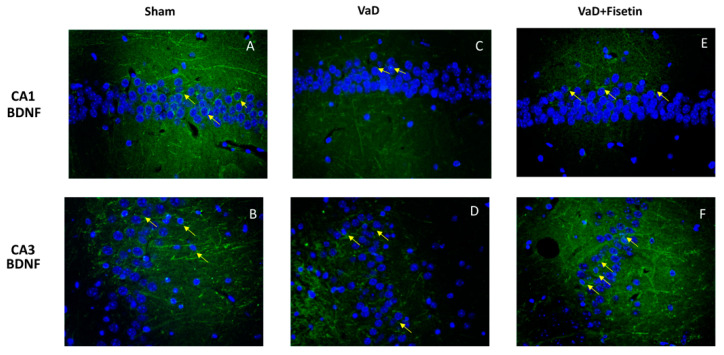
Fisetin administration on BDNF factor expression after VaD induction. Immunofluorescence for BDNF (green) in CA1 and CA3 regions in sham animals (**A**,**B**), in VaD animals (**C**,**D**), and treated with Fisetin (**E**,**F**). Figures are from at least three divided experiments.

In addition, immunofluorescence (green) for the neurotrophic factor BDNF was performed. BDNF immunoreactivity was significantly reduced in the mice of the VaD group (Figure 5C,D) compared to the sham (Figure 5A,B) while Fisetin was able to increase this immunoreactivity (Figure 5E,F).

### 3.6. Fisetin Reduced Apoptotic Process in VaD Mice

**Figure 6:**
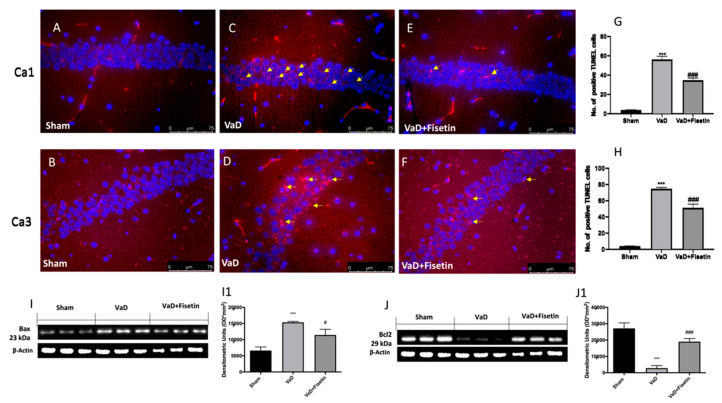
Fisetin administration on VaD induced apoptosis. TUNEL staining to see positive apoptotic cells (yellow arrows) was performed. Sham (**A**,**B**), VaD + vehicle (**C**,**D**), VaD + Fisetin (**E**,**F**). The number of apoptotic cells in CA1 and CA3 subfields of hippocampus (**G**,**H**). Figures are from at least three divided experiments. Western blots for Bax and Bcl-2 (**I**,**I1**,**J**,**J1**). Exposed is a blot of lysates (6 animals/group) with a densitometric analysis for all animals. The results = means ± SEM of 6 animals for each group. *** *p* < 0.001 vs. sham; ### *p* < 0.001 vs. VaD; # *p* < 0.05 vs. VaD. Scale bar: 75 μm. Magnification (40×).

Apoptosis has been assumed to describe cell death in a variety of neurological illnesses [25]. ROS play a serious role in apoptosis. A TUNEL assay was used to evaluate the effect of Fisetin on the apoptotic process. More apoptotic cells were found in the brains of the VaD-subjected mice (Figure 6C,D for the CA1 and CA3 region, and see graphics in Figure 6G,H) than in the sham groups (Figure 6A,B for the CA1 and CA3 region, and see graphics in Figure 6G,H). Fisetin significantly reduced the presence of apoptotic cells (Figure 6E,F for the CA1 and CA3 region, and see graphics in Figure 6G,H). The expressions of the proapoptotic BAX and anti-apoptotic Bcl-2 were detected by Western blot in the hippocampus. In the sham groups, low levels of BAX and high levels of Bcl-2 were found (Figure 6I–J1), while increased levels of BAX and reduced levels of Bcl-2 were found in the VaD-subjected animals (Figure 6I–J1). Fisetin was able to modify these levels (Figure 6I–J1).

### 3.7. Fisetin Reduced Inflammasome Pathway and Mediated IL-1β and IL-18 Release and NF-kB Expression

**Figure 7:**
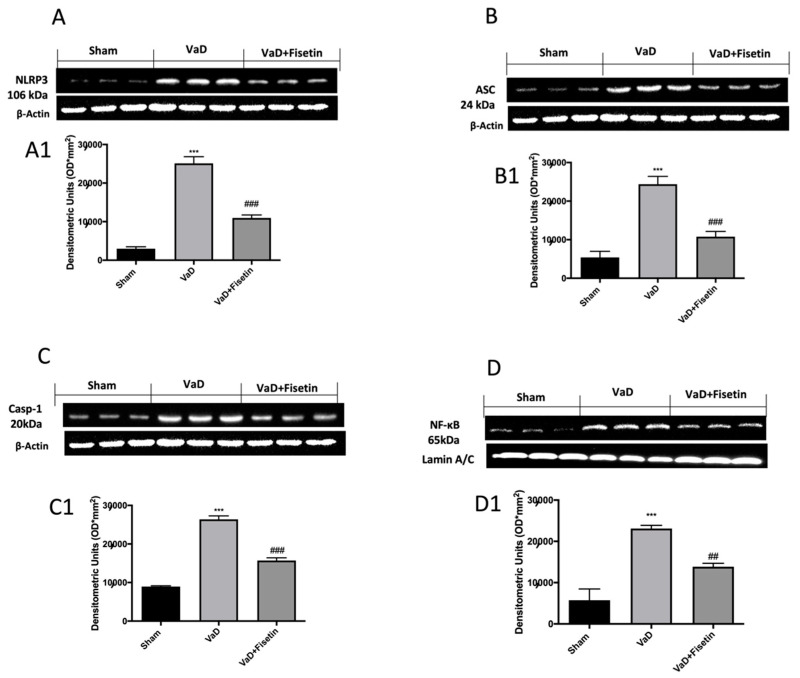
Fisetin administration on NF-κB and inflammasome activation after VaD induction. Western blots on hippocampus for (**A**) NLRP3, (**B**) ASC, (**C**) caspase 1, (**D**) NF-κB p65. Exposed is a blot of lysates (6 animals/group) with a densitometric analysis for all animals. The results in (**A1**,**B1**,**C1**,**D1**) are means ± SEM of 6 animals for each group. *** *p* < 0.001 vs. sham; ## *p* < 0.01 vs. VaD, ### *p* < 0.001 vs. VaD.

**Figure 8:**
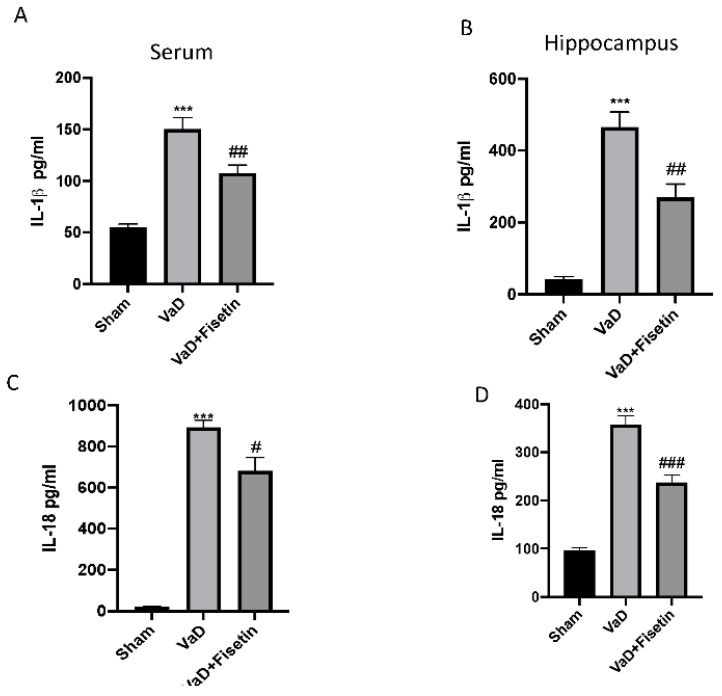
Evaluation of serum and hippocampus cytokine levels. Serum and hippocampus levels of IL-1β (**A**,**B**); serum and hippocampus levels of IL-18 (**C**,**D**). Values are means ± SEM of 6 animals for each group. *** *p* < 0.001 vs. sham; ## *p* < 0.01 vs. VaD, ### *p* < 0.001 vs. VaD, # *p* < 0.05 vs. VaD.

To better investigate whether Fisetin could act by inhibiting the inflammasome pathway, we performed a Western blot for NLRP-3, ASC, and cleaved caspase-1 as well as an ELISA Kit for IL-1β and IL-18. Compared with the control group, the expression of the NLRP3, ASC, and cleaved caspase-1 was significantly upregulated in the hippocampus tissues of the VaD mice (Figure 7A–C1). Furthermore, the ELISA results further confirmed the increased levels of IL-1β and IL-18 in the serum and hippocampus (Figure 8A–D) of the VaD animals. These data indicated that inflammasome was activated in the VaD mice. Of note, treatment with Fisetin notably inhibited the NLRP3, ASC, and cleaved caspase-1 expression as well as the IL-1β and IL-18 levels in the hippocampus of the VaD mice (Figure 7A–C1; Figure 8A–D). In addition, a Western blot for NF-κB was also performed in the hippocampal tissues. The increased nuclear NF-κB expression was observed in the VaD mice compared to the sham animals (Figure 7D,D1). Fisetin significantly reduced the level of nuclear NF-κB compared with the vehicle group (Figure 7D,D1).

### 3.8. Fisetin Oral Administration Modulated NRF-2, HO-1 in VaD Mice

**Figure 9:**
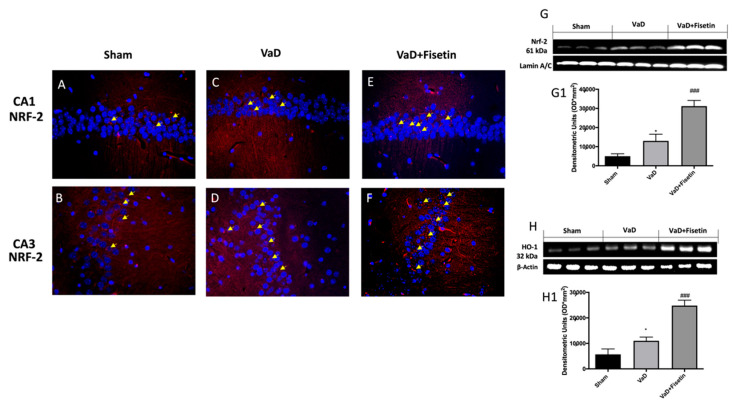
Fisetin administration on NRF2 pathway after VaD induction. Immunofluorescence for NRF-2 (red) in CA1 and CA3 regions, respectively, in sham animals (**A**,**B**), VaD animals (**C**,**D**), and VaD-subjected animals treated with fisetin (**E**,**F**). Representative Western blots on hippocampus tissues showed the effects of Fisetin on: (**G**) NRF-2, (**H**) HO-1 after VaD induction. Shown is a representative blot of lysates from 6 animals/group, together with a densitometric analysis for all animals. The results in (**G1**,**H1**) are expressed as means ± SEM of 6 animals for each group. * *p* < 0.05 vs. sham; ### *p* < 0.001 vs. VaD.

To better investigate whether Fisetin could act by promoting other signaling pathways related to inflammasome and oxidative stress, a Western blot for NRF-2/HO-1 pathways was also performed in the hippocampal tissues. The immunoreactivity (red fluorescence) of NRF-2 was physiologically increased in the VaD vehicle group (Figure 9C,D) compared to the control (Figure 9A,B), while Fisetin was able to significantly increase NRF-2 immunoreactivity (Figure 9E,F). By Western blot, a slight increase in Nrf-2 expression was observed in the VaD mice compared to the sham animals (Figure 9G,G1). Fisetin significantly upregulated Nrf-2 compared with the vehicle group, suggesting that fisetin increased Nrf-2 expression (Figure 9G,G1). At the same time, Western blot analysis showed that fisetin treatment significantly enhanced HO-1 protein expression compared to the vehicle (Figure 9H,H1).

## 4. Discussion

VaD is a disease that is caused by a decline in regional CBF and involves oxidative stress and inflammation. Neuronal cell death and brain damage are caused by increased ROS production [44]. ROS is also emerging as a key regulator of inflammasome NLRP3/NALP3 activation, which is linked to a variety of acute and chronic neurological disorders [45]. In any case, the pathophysiology of dementia is unknown, and effective treatments for the disease are absent. Fisetin (3, 3′, 4′, 7-tetrahydroxyflavone) is a polyphenolic flavone molecule found in a variety of fruits and vegetables, including strawberries [46]. Fisetin has a number of pharmacological advantages, including anti-inflammatory, anti-apoptotic, antioxidant, anti-tumorigenic, and anti-angiogenic actions, with no side effects documented even at high dosages [46]. Therefore, in the present study, repeated ischemia-reperfusion (IR) of the total bilateral carotid artery was used to establish a mouse VaD model in order to investigate the action of fisetin on NF-κB, inflammasome, as well as Nrf2/HO-1 molecular pathways. NLRP3 inflammasome is a cellular protein complex whose primary role is to trigger caspase-1, IL-1, and IL-18 synthesis [47], then process apoptosis and inflammation, aggravating brain injury [48]. This could lead to the activation of glial cells, neuronal loss, and brain circuit dysfunction. Cleaved caspase-1 may also stimulate cell death by the activation of apoptosis [9,49]. Here, we found that VaD caused brain injury, lipid peroxidation, and neuronal death in regions of the hippocampus, as well as astrocyte and microglial activation, and reduced BDNF neurotrophic factor expression together with behavioral alterations. In addition, our study divulged that repeated bilateral common carotid arteries induce the activation of inflammasome constituents (NLRP-3, ASC, and caspase 1), and their downstream products (IL-1β and IL-18) release and indorse the activation of apoptotic cell death. Fisetin attenuated histological injury, MDA levels, inflammasome pathway activation, and the mediated production of cytokines IL-1β and IL-18, apoptosis, as well as increased BDNF expression, and reduced astrocyte, microglial activation, and cognitive deficits following VaD. These results are in agreement with previous studies in which Fisetin was able to significantly reduce histological aberrations, such as neuronal degeneration, oxidative stress, necrosis, and inflammatory infiltration induced in a model of spinal cord injury as well as in a model of dementia induced by HHcy [19,26].

ROS have been proven to be a major mechanism in the production and activation of the NLRP3 inflammasome in response to a variety of external stimuli as well as endogenously produced or secreted chemicals from injured cells [37,50]. Excess ROS can have a variety of negative effects, including the activation of nuclear factor NF-κB, which leads to the increased production of pro-inflammatory cytokines. NF-κB can motivate the NLRP3 inflammasome and promote the maturation of pro-inflammatory cytokines [51]. In this study, we observed an increased expression of nuclear NF-κB in VaD mice that was reverted by Fisetin. Nrf2 and NF-κB are key regulators of the body’s response to oxidative stress and inflammation. Nrf2 plays an important role in the oxidative stress system by controlling the antioxidant response element-driven genes (ARE). Activated Nrf2 could inhibit the activation of the NLRP3 inflammasome in several diseases. Based on this, in our study, we observed physiological NRF-2 and HO-1 expressions in the CA1 and CA3 hippocampal regions in VaD mice while Fisetin treatment markedly increased the expression of NRF-2 and HO-1.

## 5. Conclusions

**Figure 10:**
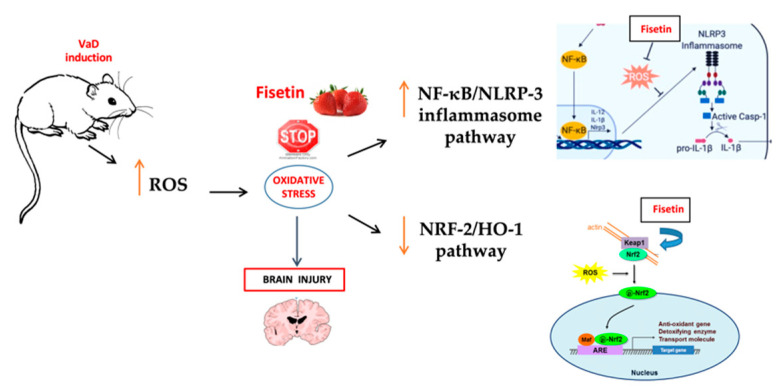
The effect of fisetin on the key molecules and respective signaling pathways.

In conclusion, our findings showed the involvement of the NF-κB/NLRP3 inflammasome signaling activation, oxidative stress, and inflammatory cytokines in VaD mice. Fisetin had a protective action with the attenuation of ROS production, inflammation, and apoptosis through its effects on the inhibition of the ROS-induced activation of the NF-κB/NLRP3 inflammasome together with the activation of Nrf2/HO-1, suggesting a possible crosstalk between these molecular pathways (Figure 10).

In addition, in this study, to induce VaD, we used a model of repetitive bilateral common carotid arteries occlusion based on the literature which could also be considered as a quasi-chronic hypoperfusion model [52]. Our results confirmed the incidence of dementia-like symptoms; however, this does not assure that all the models of VaD were comparable. Although it is well-known that VaD is mostly affected by stroke, it can also be provoked by hypoperfusion. However, the mechanisms relative to cognitive dysfunction due to hypoperfusion are not well comprehended and model animals are frequently used to study VaD resulting from hypoperfusion. Nevertheless, there are many questions elevated in research linking stroke and dementia which are generally unanswered. It is critical to apprehend early events of stroke and stroke leading to VaD. Very little can be done after disease onset starts and therapies need to be started as soon as possible, with its direct objective being to regularize perfusion and to mediate any biochemical disfunction to recuperate the damage as early as possible.

## Data Availability

For a rule of our laboratory the datasets used in the current study are available from the corresponding author (rsiracusa@unime.it) on reasonable request.
